# Fatigue Response of MoS_2_ with Controlled Introduction of Atomic Vacancies

**DOI:** 10.1021/acs.nanolett.3c02479

**Published:** 2023-11-16

**Authors:** Yolanda Manzanares-Negro, Aitor Zambudio, Guillermo López-Polín, Soumya Sarkar, Manish Chhowalla, Julio Gómez-Herrero, Cristina Gómez-Navarro

**Affiliations:** † Departamento de Física de la Materia Condensada, Universidad Autónoma de Madrid, Cantoblanco 28049, Spain; ‡ Departamento de Física de Materiales, Universidad Autónoma de Madrid, Cantoblanco 28049, Spain; § Department of Materials Science and Metallurgy, University of Cambridge, CB30FS Cambridge, U.K.; ∥ IFIMAC, Universidad Autónoma de Madrid, Cantoblanco 28049, Spain

**Keywords:** 2D materials, TMDCs, fatigue, strain, sulfur vacancies, nanoindentations

## Abstract

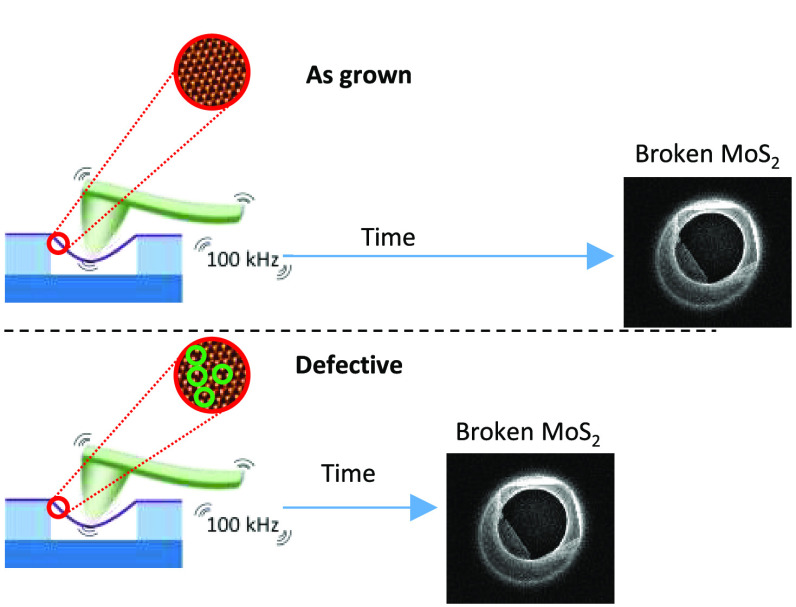

Fatigue-induced failure resulting from repetitive stress–strain cycles is a critical concern in the development of robust and durable nanoelectromechanical devices founded on 2D semiconductors. Defects, such as vacancies and grain boundaries, inherent in scalable materials can act as stress concentrators and accelerate fatigue fracture. Here, we investigate MoS_2_ with controlled atomic vacancies, to elucidate its mechanical reliability and fatigue response as a function of atomic defect density. High-quality MoS_2_ demonstrates an exceptional fatigue response, enduring 10^9^ cycles at 80% of its breaking strength (13.5 GPa), surpassing the fatigue resistance of steel and approaching that of graphene. The introduction of atomic defect densities akin to those generated during scalable synthesis processes (∼10^12^ cm^–2^) reduces the fatigue strength to half the breaking strength. Our findings also point toward a sudden defect reconfiguration prior to global failure as the primary fatigue mechanism, offering valuable insights into structure–property relationships.

The mechanical endurance of materials is typically limited by their ultimate breaking strength or lifetime due to fatigue induced by cyclic loading at stress below their ultimate tensile strength. Notably, over 80% of fracture incidents occur as a result of fatigue.^1^ The achievement of a sustainable future requires the use of durable materials that can withstand repeated mechanical stress. Two-dimensional (2D) materials such as graphene and transition metal dichalcogenides (TMDCs), particularly MoS_2_, are being investigated as active components in a variety of electromechanical devices, i.e., flexible displays, mechanical sensors, and nanomechanical resonators,^2,3^ due to their unique electronic and mechanical properties, such as appropriate band gap, exceptional electrostatic gate coupling, high flexibility, and ultrahigh strength. While graphene and MoS_2_ have been widely employed to enhance the fatigue resistance of bulk materials and structures,^4−7^ experimental works into the service life of atomic thin layers have only been conducted in recent years,^8,9^ owing to the challenges of performing such experiments. However, with the increasing adoption of few-layered devices in practical applications, their mechanical reliability and service life have become critical concerns.

Similar to bulk materials, the presence of defects, such as atomic vacancies, substitutional atoms, or grain boundaries, modifies the mechanical response of these materials, usually decreasing their intrinsic strength.^10−12^ Unfortunately, every scalable method for the production of these materials involves a certain (usually low) density of imperfections in their atomic lattice. Therefore, systematic studies on physical magnitudes upon defect content should enlighten the tolerance of these materials in the road to real life applications.^13^

Here we evaluate the mechanical reliability and fatigue response of MoS_2_ by means of indentations with atomic force microscopy (AFM) on suspended membranes. By analyzing multiple breaking events, we determine that monolayered MoS_2_ has a reliability similar to engineered ceramics. We demonstrate that the dynamic fatigue life of high-quality CVD grown monolayered MoS_2_ is greater than 10^9^ cycles for a stress value of 13.5 GPa, which is 0.8 times its ultimate breaking strength. Upon the controlled introduction of atomic vacancies, we perform a systematic study of these magnitudes as a function of defect density. Lateral force microscopy images before and after fatigue testing of the membranes reveal that fatigue results from a sudden defect reconfiguration prior to global failure.

We performed fatigue measurements on MoS_2_ monolayer drumheads with diameters ranging from 0.5 to 2 μm. Our starting MoS_2_ monolayers were grown by CVD (see SI1) and then transferred by an all-dry technique^14^ onto SiO_2_/Si substrates with predefined micrometric circular wells yielding suspended membranes of MoS_2_ well anchored on the circular perimeter.^15^ We confirmed the presence of the MoS_2_ monolayers using photoluminescence microscopy^16^ (data provided in SI2) and imaged them using AFM in dynamic mode. For this study, we selected only single-layer drumheads exhibiting no observable slack or wrinkling (Figure 1a). We estimated the defect density using micro-Raman spectroscopy (details in SI2).^11,17,18^ We obtained native defect densities of 0.4 × 10^12^ and 0.25 × 10^12^ cm^–2^ for two different batches of as-grown samples, corresponding to mean distance between defects of ⟨*l*_d_⟩ = 15.7 nm and ⟨*l*_d_⟩ = 20 nm, respectively. These defect densities are typical of ultra-high-quality MoS_2_ CVD grown samples.^19^

**Figure 1:**
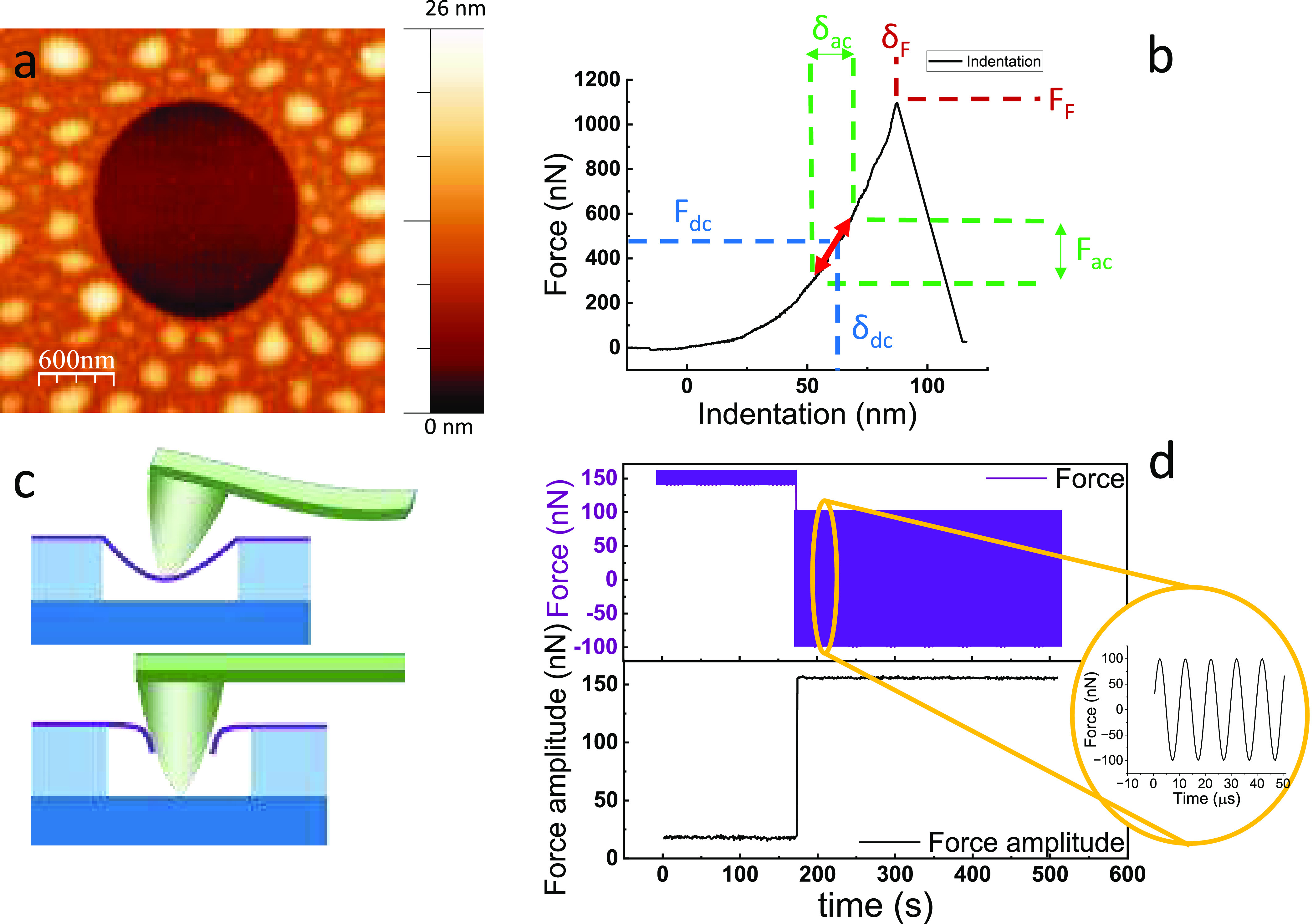
(a) AFM image of a representative MoS_2_ microdrum. (b) Force vs indentation curve on a MoS_2_ microdrum where the DC and AC forces are marked, along with the corresponding indentation. (c) Upper panel: illustration of an AFM tip indenting a microdrum. Lower panel: sharp decrease in the deflection of the cantilever at the fracture point. (d) Representative data observed near the fracture point. Before failure, the cantilever amplitude is low as a consequence of the reacting force of the suspended membrane. After failure, the cantilever amplitude increases with the free oscillation amplitude.

Experiments were performed under ambient conditions (21 °C and ∼30% humidity) using a custom-made AFM. Prior to fatigue testing, we conducted regular indentations at the center of the drumheads. From these indentation curves, we calibrated the force and indentation as shown in Figure 1b and discarded slippering of the MoS_2_ at high loading force. We also estimated the elastic modulus of the MoS_2_ layers and the residual stress in the membranes yielding values of 200 and ∼0.15 N/m, respectively (see SI3 for details). Subsequently, we applied a static force (with a corresponding static stress σ_DC_) at the center of the suspended membrane and oscillated the AFM probe at a prefixed amplitude around the static load at a frequency of 100 kHz, inducing a dynamic stress σ_AC_. We maintained these conditions until fracture. We detected fatigue failure by observing an abrupt increase in the cantilever deflection and a sudden increase in the cantilever amplitude, as shown in Figure 1c,d. We confirmed the membrane failure using AFM images acquired after this event (SI4 fatigue protocol).

Prior to conducting fatigue tests, we indented numerous as-grown MoS_2_ monolayer drumheads until they fractured. This allowed us to determine the fracture force of the membranes. Then, we estimated the ultimate breaking strength σ_F_ using^20^
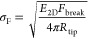
1where *E*_2D_ is the two-dimensional Young’s modulus, *F*_break_ is the fracture force, and *R*_tip_ is the radius of the indentation tip. This expression ignores nonlinear elasticity, and the derived value is known to overestimate the strength by about 10%; however, it has widely been used in the literature.^21,22^ Our measurements yielded an average breaking strength of ⟨σ_F_⟩ = 17 ± 1 GPa, with no dependence on the residual stress of each membrane (data in SI5), consistent with previous studies.^11,23^

In what follows, all strength values will be normalized to the average ultimate breaking strength of as-grown monolayered MoS_2_, i.e., 17 GPa. As shown in Figure 2a, our data are well described by a two-parameter (*m*, ⟨σ_F_⟩) nanoscale Weibull distribution^24,25^
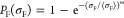
2where *σ*_F_ is the fracture strength measured for each indentation and ⟨σ_F_⟩ is the average value for all *σ*_F_ measured in the experiments.

**Figure 2:**
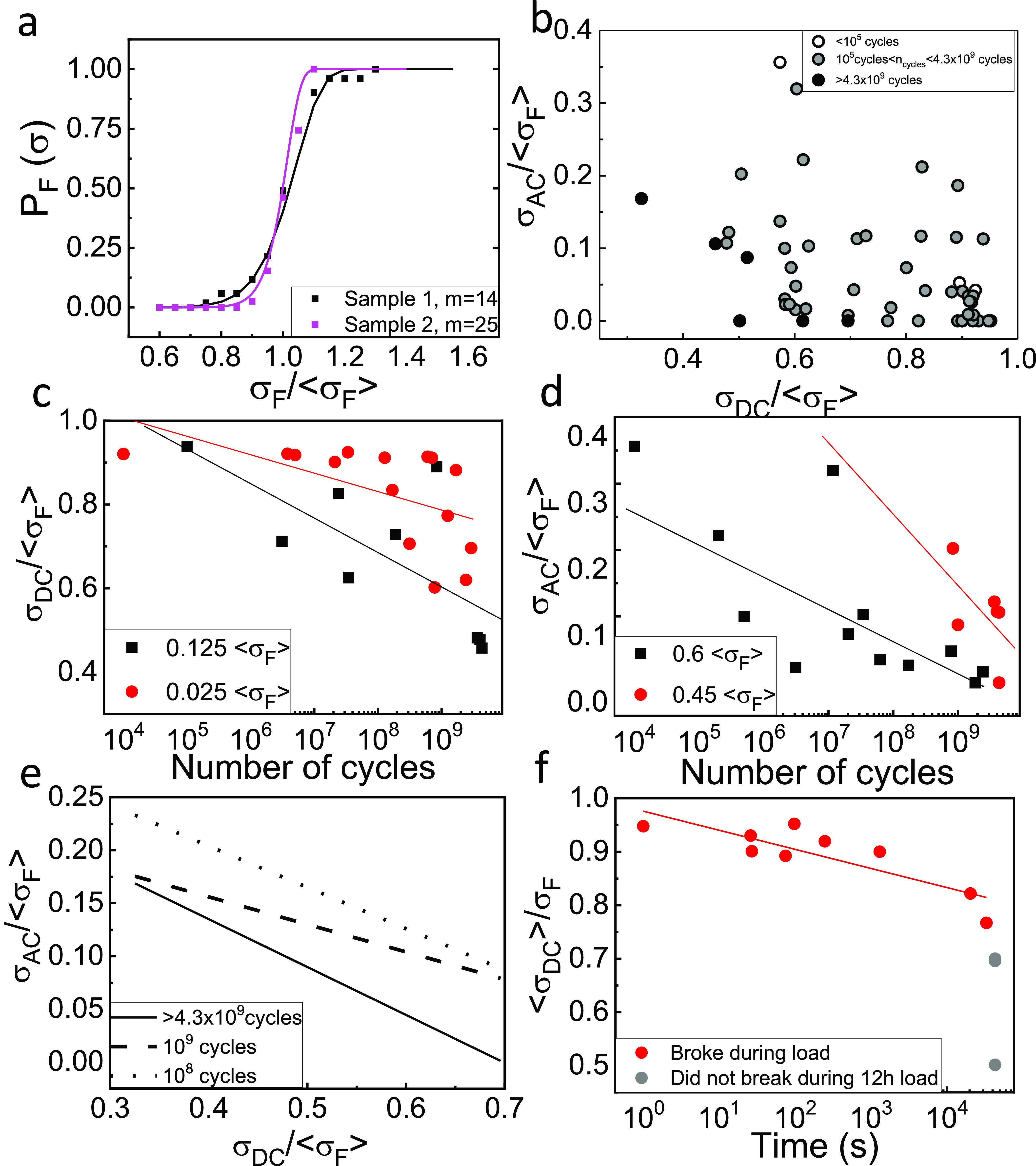
(a) Probability plot of the surveillance of MoS_2_ drumheads at different stresses and corresponding Weibull fitting for the two batches of as-grown samples. (b) Goodman diagram representing the applied static (horizontal axis) and dynamic (vertical axis) stress, normalized to the mean breaking strength. Black circles correspond to drumheads that survived after 4.3 × 10^9^ cycles, gray circles represent those that fractured between 1 and 4.3 × 10^9^ cycles, and white dots represent those that broke just after reaching the DC load. (c) S–N diagram with varying σ_DC_ at two different σ_AC_ of 0.125⟨σ_F_⟩ and 0.025⟨σ_F_⟩. (d) S–N diagram of microdrums supporting σ_AC_/σ_F_ of 0.6⟨σ_F_⟩ and 0.45⟨σ_F_⟩ with varying σ_AC_. (e) Goodman lines for microdrums that did not fracture (solid line, lim = 0.45⟨σ_F_⟩, *c* = 1.5), for 10^9^ cycles (dashed line, lim = 0.26⟨σ_F_⟩, *c* = 1) and 10^8^ cycles (dotted line, lim = 0.36⟨σ_F_⟩, *c* = 1.1). Note that, for nonbroken microdrums, we only provide an upper limit. (f) Static fatigue for as-grown MoS_2_. Red dots: microdrums that broke in times lower than 12 h. Gray dots: microdrums that did not break after 12 h of static loading.

We characterized two batches of as-grown drumheads with the above-mentioned native defect densities finding Weibull modulus values of *m* = 14 and *m* = 25, for ⟨*l*_d_⟩ = 15.7 nm and ⟨*l*_d_⟩ = 20 nm, respectively. The Weibull modulus describes the variability in material strength, and in bulk materials, it is used as an indicator of mechanical reliability. Although the direct applicability of this analysis to nanostructures still has some limitations (detailed discussion in SI6), we compared our results to those reported previously. Our Weibull modulus is lower than the typical values for metals (*m* ∼ 100) and that reported for graphene (*m* ∼ 16–44).^8,21^ However, it is higher than that of the best-engineered ceramics (*m* ∼ 10) and similar to that reported for as-grown MoS_2_ in a very recent study (*m* ∼ 22).^26^

We performed fatigue characterization by applying *σ*_DC_ and *σ*_AC_ and measuring the number of cycles for drumhead survival before failure. We used Goodman diagrams to visualize fracture statistics. Figure 2b shows our data of as-grown monolayer MoS_2_, where black dots represent the membranes that did not break after 4.3 × 10^9^ loading cycles and white dots represent those that failed right after reaching the load conditions. From this plot, we extracted stress-number of cycles (S–N) graphs performed at a constant *σ*_AC_ and varying *σ*_DC_ and at a constant *σ*_DC_ and varying *σ*_AC_. These results are depicted in Figure 2c,d, respectively, where the fatigue life of MoS_2_ is shown to be strongly dependent on both *σ*_DC_ and *σ*_AC_.

Our data reveal a fatigue strength of 0.8⟨σ_F_⟩ for 10^9^ cycles. These results place high-quality CVD grown MoS_2_ as one of the best materials in terms of dynamic fatigue response, with a high level of survival, 1 order of magnitude higher than those of high-strength steels in absolute and relative terms. The best alloys show a fatigue endurance of about 0.5⟨σ_F_⟩, corresponding to 0.5 GPa for the case of steel. As-grown MoS_2_ also exceeds by far the fatigue lifetime of other nanostructures such as Si nanobeams..^27,28^ Comparable values to those reported here have been recently reported only for graphene.^8^ It is worth noting that our normalized S–N plots superpose those of graphene (see SI7).

We can also define Goodman lines from our data. These lines define the regions where the membranes do not break after a certain number of fatigue cycles and are commonly expressed as^29^
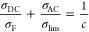
3where the parameter *σ*_lim_ is the maximum *σ*_AC_ that the material can withstand without breaking, when *σ*_DC_ = 0. Additionally, *c* is known as the safety factor, which indicates how many times a component is safer than what is required for a given use.^30^ For the case of 4.3 × 10^9^ cycles, we obtain *σ*_lim_ = 0.45⟨σ_F_⟩ (7.7 GPa) and *c* = 1.5. Goodman lines for 4.3 × 10^9^, 10^9^, and 10^8^ cycles, as depicted in Figure 2e, show a very high tolerance to a large number of cycles, a characteristic that is only achieved by metal alloys.

Scanning electron images of the membranes fractured by fatigue tests showed micrometer length tears with straight and sharp edges (starting at the center of the drumhead and reaching the walls of the wells) and crack propagation along crystallographic directions, indicating global and catastrophic failure (Images provided in SI8). For those drumheads that survived 4.3 × 10^9^ cycles, AFM topography images after fatigue testing did not show any evident change. Moreover, subsequent indentations also depicted similar breaking strength and elastic response to the nonirradiated membranes. Since the strength of two-dimensional materials is highly dependent on the size of defects,^11^ this result suggests that the dimension of flaws in the most strained region (under the tip) rarely undergo significant alterations during the fatigue process and point toward an abrupt atomistic mechanism of fatigue without progressive damage. It also poses dynamic fatigue proof testing as a noninvasive technique as an approach for high reliability sample selection.

We expanded our dynamic fatigue study to incorporate static loading conditions, which is a key factor in determining the service life of materials. These results are included in Figure 2f.

In classical fracture mechanics, fatigue cracks start at the site of the highest local stress in a device.^1^ In macrostructures, this usually happens at the holes or notches. In microstructures, these are inclusions, voids, cavities, or scratches. For highly crystalline atomically thick materials such as TMDCS, imperfections in the atomic lattice are the expected root cause of fatigue initiation. The most common atomic defect in MoS_2_ are single sulfur vacancies,^19,31,32^ which are inherent in any large-scale production method due to their low defect formation energy.^33^ Single sulfur vacancies reduce the strength of MoS_2_ and increase fracture toughness.^11^ However, the influence of defects on the fatigue lifetime is still unexplored. In what follows, we report our results on this topic.

We produced MoS_2_ samples with a controlled type and defect density by irradiating samples with doses of Ar^+^ at 500 eV with perpendicular incidence at different irradiation doses. The techniques used to characterize the samples are described in our previous study^11^ and SI2. Summarizing, irradiation generated homogeneous densities of vacancies, mainly sulfur monovacancies (∼80% of created defects), and a smaller percentage of single Mo vacancies and double sulfur vacancies. Consecutive doses resulted in higher defect densities. We estimated defect densities of 0.4 × 10^12^ cm^–2^ for the as-grown sample and 1.4 and 2.4 × 10^12^ cm^–2^ for two consecutive irradiations, corresponding to mean defect distances of ⟨*l*_d_⟩ = 15.7 nm, ⟨*l*_d_⟩ = 8.6 nm, and ⟨*l*_d_⟩ = 6.5 nm, respectively.

Fatigue response of samples with controlled densities of atomic vacancies is depicted in Figure 3. To enable direct comparison, we also included the results for as-grown MoS_2_ in these plots. Figure 3a shows that the ultimate breaking strength of the irradiated samples decreased from 17 GPa for the as-grown samples to 10 and 9.7 GPa for the two consecutive irradiation doses, as previously reported.^11^ Weibull plots in Figure 3b, show that introduction of atomic vacancies decreases reliability by decreasing Weibull modulus from *m* ∼ 14 for the as-grown samples to *m* ∼ 11 and *m* ∼ 8 for the two irradiated batches of samples, respectively, showing a clear trend. However, it should be noted that the observed decrease in Weibull modulus measured by nanoindentations cannot be directly extrapolated to globally stressed samples (see SI6 for a detailed discussion).

**Figure 3:**
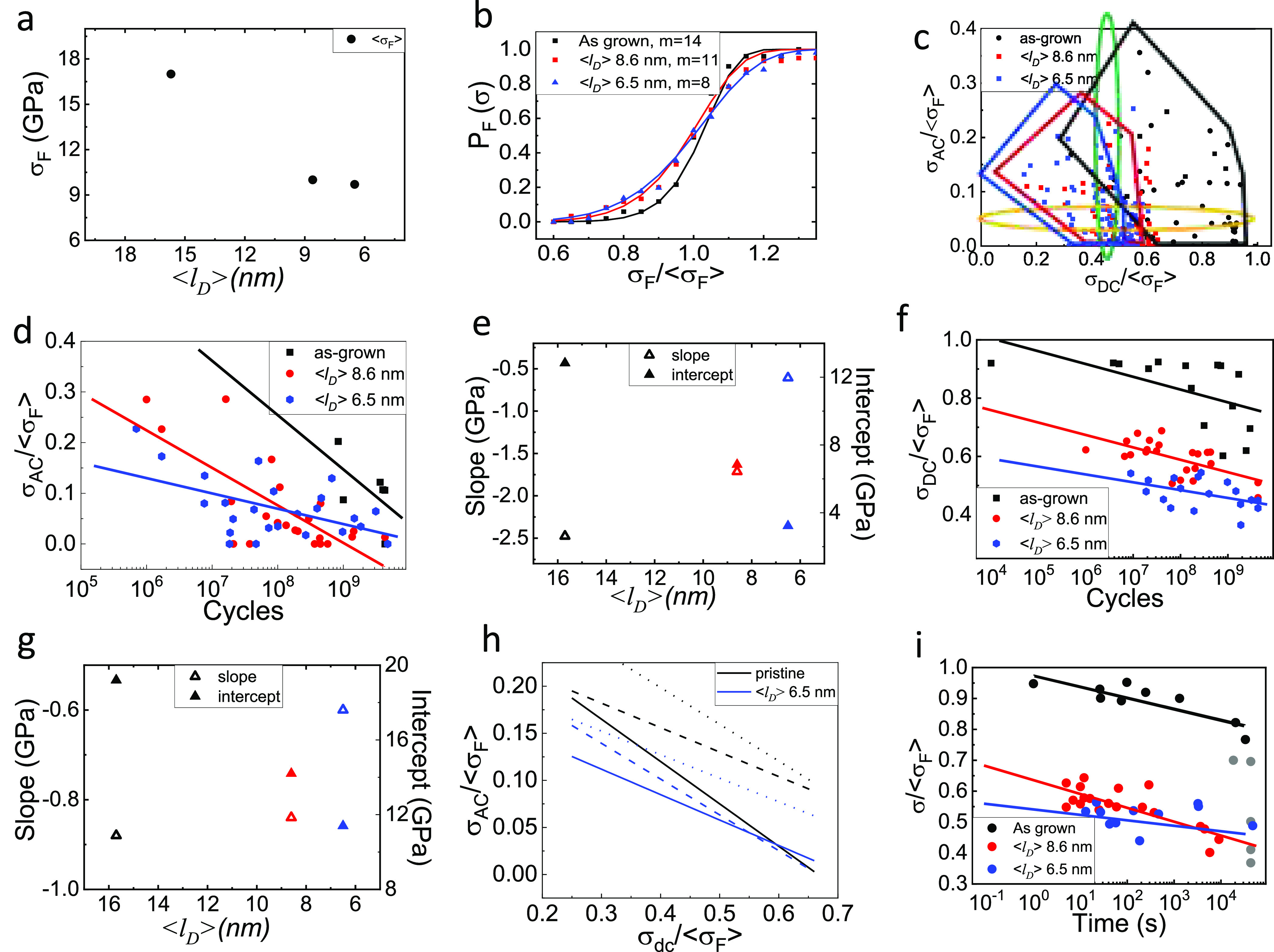
(a) Breaking strength as a function of mean distance between defects. (b) Survival probability and Weibull fitting for as-grown drumheads and those with two consecutive irradiation doses. (c) Goodman diagram showing all results in as-grown and irradiated drumheads. Green and yellow regions indicate the data selected for plots in panels d and f, respectively. (d) Data obtained with a constant σ_DC_ of 0.45⟨σ_F_⟩ and varying *σ*_DC_. (e) Intercept and slope of the linear fittings in panel d. (f) Data obtained with a constant *σ*_AC_ of 0.05⟨σ_F_⟩ and varying *σ*_DC_. (g) Intercept and slope of the linear fittings in panel f. (h) Goodman lines for drums that did not break (solid line), those that failed at 10^9^ cycles (dashed lines), and microdrums fractured at 10^8^ cycles for as-grown (black) and irradiated sample with ⟨**l**_d_⟩ = 6.5 nm (blue). (i) Static fatigue lifetime of microdrums with different defect densities. Solid lines are linear fits to the data of the coresponding color.

The Goodman diagram in Figure 3c summarizes the results of dynamic fatigue tests conducted on both pristine and irradiated drumheads. The measurements obtained with constant *σ*_DC_, enclosed in the green ellipse of Figure 3c, are presented in Figure 3d, with similar plots available in SI9. Linear fits are drawn as continuous lines. Despite the dispersion of experimental data in irradiated samples due to the stochastic nature of brittle failure in MoS_2_, the representation of slopes and intercepts with the *y*-axis of linear fits in Figure 3d reveals a robust trend, as shown in Figure 3e. The *y*-axis intercept indicates the maximum sustainable value of *σ*_AC_, which decreases from 12 to 6 and 3 GPa as the defect density increases. The slope reflects the sensitivity of fracture strength to the number of cycles or the change in survival stress per order of magnitude in the number of cycles. Figure 3f illustrates the lifespan of the samples with a fixed *σ*_AC_ of 0.125⟨σ_F_⟩ (encircled by the yellow ellipse in Figure 3c). It is evident from the plot that the survival cycles at a constant *σ*_AC_ decrease with increasing defect density. Again, the slopes and *y*-axis intercepts are presented in Figure 3g, demonstrating robust trends, as seen in Figure 3e,g. These trends permit the extrapolation of the fatigue response for densities of atomic vacancies within the standard range for MoS_2_ produced by scalable methods. Interestingly, we did not observe a fatigue endurance neither for pristine nor for defective drumheads.

Based on our results, the fracture strength at 10^9^ cycles appears to be a suitable comparison point for samples with varying densities of atomic vacancies. As-grown MoS_2_ displays a fatigue strength of 0.8⟨σ_F_⟩ (*σ*_DC_ = 13.6 GPa, *σ*_AC_ = 0.45 GPa), which decreases to 0.6⟨σ_F_⟩ (*σ*_DC_ = 10.2 GPa, *σ*_AC_ = 0.45 GPa) and 0.5⟨σ_F_⟩ (*σ*_DC_ = 8.5 GPa, *σ*_AC_ = 0.45 GPa) with the introduction of 1.4 × 10^12^ and 2.4 × 10^12^ cm^–2^ of atomic defect densities, primarily sulfur vacancies. This line of reasoning allows for the plotting of Goodman lines to define safety regions for both as-grown and irradiated samples. Figure 3h illustrates these lines for the as-grown and most irradiated sample. In the case of the irradiated sample with 2.4 × 10^12^ cm^–2^ (⟨*l*_d_⟩ = 6.5 nm), Goodman lines at 4.3 × 10^9^ cycles yield σ_lim_ = 0.27 (4.6 GPa) and *c* = 1.4. These safety lines, even for irradiated samples, demonstrate the high fatigue resistance of MoS_2_ relative to the best bulk materials, such as high-strength steel. Goodman lines for steel at 10^7^ cycles are in the range of hundreds of MPa, implying an improvement of 2 orders of magnitude in the number of cycles and 1 order of magnitude in typical stresses.

In Figure 3i, we observe a trend of diminishing breaking strength over extended periods of static loading with decreasing breaking stresses for increasing defect density. This observation points out the influence of thermal fluctuations under ambient conditions, which mimic the effects of small-amplitude stress cycles but at a much higher frequency. To draw a comparative perspective, considering a characteristic phonon frequency of 10^13^ Hz for thermal fluctuations, for a given σ_DC_/⟨σ_F_⟩ achieving membrane failure due to thermal fluctuations would require 10^8^ cycles more than those induced by *σ*_AC_ = 0.05⟨σ_F_⟩, as expected for picometer-sized fluctuation caused by phonons at room temperature. This outcome aligns with prior research, corroborating that thermal fluctuations, while exerting a lesser impact than induced cycling, can indeed contribute to the rupture of covalent bonds when subjected to applied stress levels below the fracture threshold.^8,26^

The failure time τ for a material under an applied stress was described decades ago for polymers by Zurkhov et al.^34^ using the qualitative relation , where τ_0_ is the reciprocal of the natural frequency of the atoms (about 10^13^ Hz), *U*_0_ is the average energy required to break atomic bonds, and γ is a coefficient that translates stress to energy and proportionally decreases with the disorder. By fitting the data to this expression, we obtained an average binding energy of 190 kJ/mol for the as-grown samples, which is comparable to the value of 160 kJ/mol for sulfur bonds in bulk MoS_2_, suggesting that this empirical model can also be extrapolated to covalent materials. As expected, we also found that γ and *U*_0_ decrease with an increase in defect density.

Scanning electron microscopy images of irradiated samples after fatigue failure also showed tears propagating to the edge of the wells. A batch of samples was subjected to a fatigue test almost reaching their expected failure conditions, according to graphs depicted in Figure 3d,f. These membranes did not show changes in elastic response neither obvious topographic change after fatigue testing. However, regular AFM topographic images acquired in dynamic mode do not provide enough resolution to resolve atomic scale processes. To gain further insight into the atomistic mechanism of fatigue, we also performed lateral force microscopy (LFM) images of some membranes before and after fatigue testing (see SI10 for conditions). LFM has been shown to resolve single atomic defects when applied to 2D materials;^35^ vacancy-type defects in MoS_2_ appear in LFM images as high frictional regions. Figure 4 panels a and b depict LFM images of a MoS_2_ drumhead with an induced density of defects of 1.4 cm^2^ (i.e., ⟨*l*_d_⟩ = 8.6 nm), where defects appear as darker regions in the image. This membrane was subjected to fatigue testing approaching their expected failure conditions (5 × 10^6^ cycles with *σ*_DC_ = 0.7⟨σ_F_⟩ and *σ*_AC_ = 0.2⟨σ_F_⟩), and subsequently imaged again by LFM in the same conditions and using the same AFM probe, within a time interlap of few hours after fatigue testing. For this membrane we did observe at least one detectable change. As highlighted in Figure 4c,d, after fatigue testing, we observed a dark feature that revealed the emergence of a multiatomic defect. Among the seven membranes measured using this protocol, only one of them (that depicted in Figure 4) showed detectable changes. Although LFM can provide atomic resolution and resolve individual vacancies in small images, it is very difficult to account for these defects across the entire suspended membrane. Darker regions in these images are likely double sulfur, or molybdenum vacancies rather than sulfur monovacancies. Despite this issue, our results support previous molecular dynamic simulations^8^ where failure upon fatigue in graphene is shown to be preceded by stress mediated bond reconfiguration at vacancy defects and clustering of atomic vacancies into multiatomic ones. The fact that we only observe these changes in a reduced number of membranes also supports the idea of an abrupt atomistic mechanism of fatigue, very different from the progressive damage observed in conventional materials.

**Figure 4:**
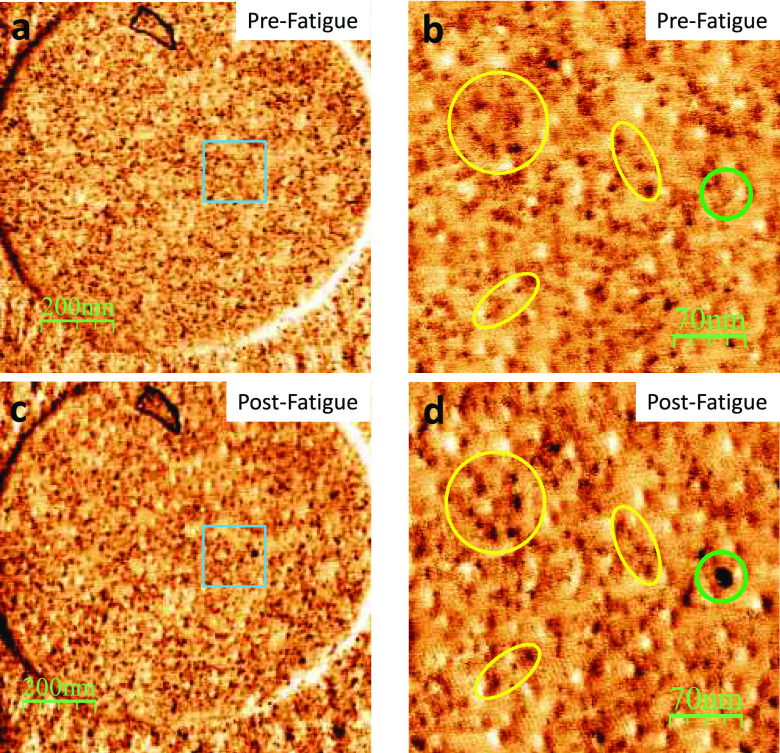
(a) 1024 × 1024 pixel LFM image of a MoS_2_ drumhead with a defect density of 1.4 cm^2^ (i.e., ⟨*l*_d_⟩ = 8.6 nm). (b) LFM of the region marked with a blue square in panel b. Yellow ellipses encircle regions that allowed localizing the desire region before and after fatigue testing and highlight regions where changes were not observed. Green circle guides the eye where the change were observed. (c) 1024 × 1024 LFM image of the same drumhead shown in panel a but after performing fatigue testing for 4 × 10^6^ cycles with *σ*_DC_ of 0.7⟨σ_F_⟩ and *σ*_AC_ of 0.2⟨σ_F_⟩. (d) LFM of the region marked with a blue square in panel c. As every scanning probe microscopy, LFM images are highly dependent on the precise atomic status of the tip apex; this accounts for slight deviations between pre- and postfatigue images (usually changes in contrast and position of the features). However, the emergent darker region comparing between images in panels c and d cannot be ascribed to tip changes.

Very recent molecular dynamic simulations concluded that the reliability of MoS_2_ results from a cooperative effect of three major ingredients: defect configuration, defect density, and thermal fluctuations.^26^ Our results quantify the influence of the density of atomic defects and point toward a non-negligible influence of thermal fluctuations upon static loading. The influence of defect configuration cannot be directly derived from the present results, but we envision creation of different kind of atomic defects, such as multivacancy, by Ga irradiation under a field ion beam,^11^ or controlled passivation of atomic vacancies^36^ to further explore the relevance of defect configuration.

Summarizing, by means of nanomechanical indentations with an AFM tip, we evaluated the mechanical reliability, dynamic and static fatigue lifespan, and safety regions of monolayered MoS_2_. A controlled introduction of atomic vacancies allowed a systematic study of these magnitudes as a function of defect content. We observe that the mechanical reliability of MoS_2_ decreases with defect induction. Dynamic fatigue testing places MoS_2_ as one of the best materials showing ultrahigh dynamic fatigue strength, with a strain tolerance at 10^9^ cycles and fatigue safety lines achieved only before by graphene and metal alloys. This tolerance decreases with defect introduction, but even the most defective samples evaluated here yet exhibit a fatigue response and safety lines comparable to metal alloys. We also provide insights into the atomistic mechanism of fatigue indicating sudden atomic reconfiguration before global failure. The results presented here, together with previous works reporting improved fracture toughness with controlled defect creation,^11^ provide a clear understanding of how atomic defects in monolayer MoS_2_ influences its mechanical resilience.

## Supporting Information Available

SI1, CVD growth of MoS_2_; SI2, photoluminescence and Raman spectra of MoS_2_; SI3, elastic characterization by nanoindentation prior to fatigue testing; SI4, fatigue protocol; SI5, dependence of breaking strength with prestress of the samples; SI6, Weibull distribution; SI7, comparison of fatigue response of MoS_2_ and graphene; SI8, scanning electron microcopy images of MoS_2_ membranes fractured by fatigue; SI9, complementary S–N plots; SI10m lateral force microscopy details (PDF)
