# Supplementary material for: Fatigue Response of MoS2 with Controlled Introduction of Atomic Vacancies
Source: Nano Lett. 2023 Nov 16;23(23):10731–8. doi: 10.1021/acs.nanolett.3c02479 (PMC10722543; doi:10.1021/acs.nanolett.3c02479)
Supplement: Supplementary file 1 — nl3c02479_si_001.pdf [file nl3c02479_si_001.pdf]

Supplementary Information

Fatigue response of MoS_2 with Controlled Introduction of Atomic Vacancies

Yolanda Manzanares-Negro[1], Aitor Zambudio[1], Guillermo López-Polín[2], Soumya Sarkar[3], Manish Chhowalla[3], Julio Gómez-Herrero[1,4], Cristina Gómez-Navarro[1,4].*

[1] Departamento de Física de la Materia Condensada, Universidad Autónoma de Madrid, Cantoblanco 28049, Spain
[2] Departamento de Física de Materiales, Universidad Autónoma de Madrid, Cantoblanco 28049, Spain
[3] Department of Materials Science and Metallurgy, University of Cambridge, CB30FS Cambridge, UK
[4] IFIMAC, Universidad Autónoma de Madrid, Cantoblanco 28049, Spain
* cristina.gomez@uam.es

Supplementary Information 1: CVD growth of MoS_2

For the growth of MoS_2 a single-zone tube furnace was used. 2.5 mg MoO_3 powder was evenly distributed in an alumina boat and located at the center of the furnace. A layer of molecular sieve (Alfa Aesar 5A 1-2 mm diameter pellets) covered the MoO_3 boat to control the growth rate. SiO_2/Si substrates, spin-coated with 0.5 mg/mL NaOH promoter, were placed on the MoO_3 boat. 60 mg sulfur powder in another alumina boat was located 17 cm upper stream at the edge of the furnace. Before starting growth, the tube was purged with 460 sccm N_2 for 20 minutes. The N_2 flow rate was then decreased to 60 sccm. The temperature of MoO_3 was set and kept at 720 °C for around 10 minutes, while the temperature of sulfur could reach 230 °C. At the finishing step, the furnace was switched off, the sulfur source was pulled out of the heating zone, and the MoO_3 boat was let to cool down in 460 sccm N_2 gas.

Supplementary Information 2: Photoluminescence and Raman spectra of MoS_2

Identification of monolayerMoS_2:

MoS_2 single layers were identified by Photoluminescence (PL) spectroscopy. As reported previously, the PL of MoS_2 is highly enhanced on single layers.[1] This effect becomes even greater when the single layers are suspended, as the micro drums studied in this article. We use this enhanced photoluminescence to better identify the suspended single layers of MoS_2. The PL spectra for a suspended single layer, a single layer on substrate and bulk MoS_2, as well as an image of the intensity contrast in a region with several micro drums, are depicted on **Figure S1**.

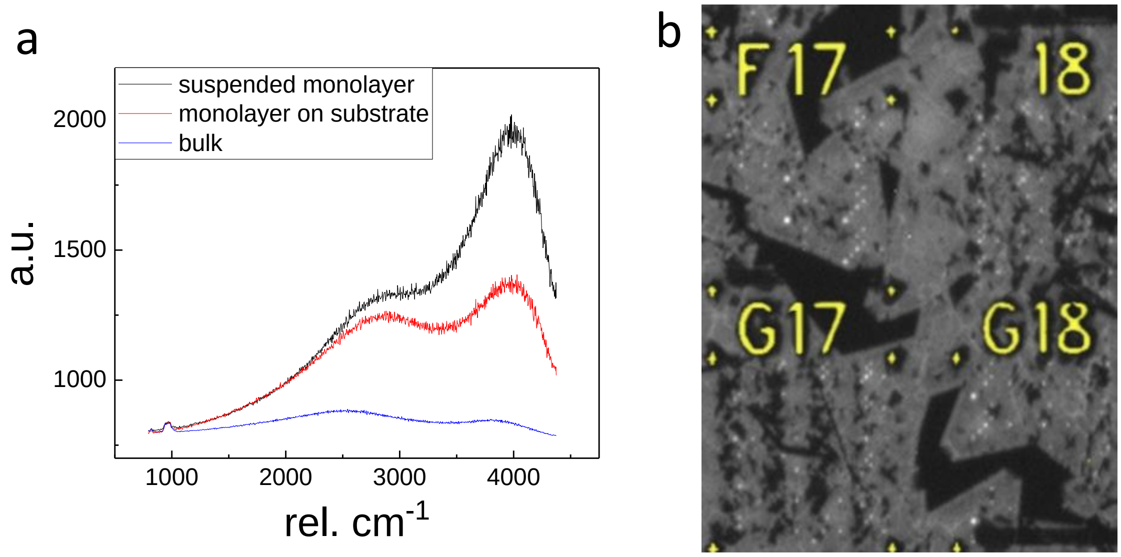

Figure SI1. a)PL espectra for a suspended MoS_2 monolayer, a MoS_2 monolayer supported by a substrate and bulk MoS_2. The three different regions present high variations in the intensity.b) PL image of a region presenting several microdrums. White circles correspond to microwells covered by suspended MoS_2 single layers, while the surrounding grey areas are the single layers supported by the substrate. Black regions correspond to areas where the covering MoS_2 flake is thicker (2 or more layers) and yellow numbers are substrate markers.

Defect characterization from Raman Spectra

The Raman spectra of MoS_2 presents two prominent peaks, E' (382 cm-1) and A_1' (407 cm-1). The presence of defects causes the broadening of those peaks, as well as the arising of a new peak, *LA,* at 227 cm^{-1}.[2–4] In the case of our samples, with a very low defect density, this peak is especially difficult to identify. In **Figure S2** we can observe the Raman spectra for the as-grown sample and the most irradiated sample used for this work, and a zoom in the region around the emerging peak.

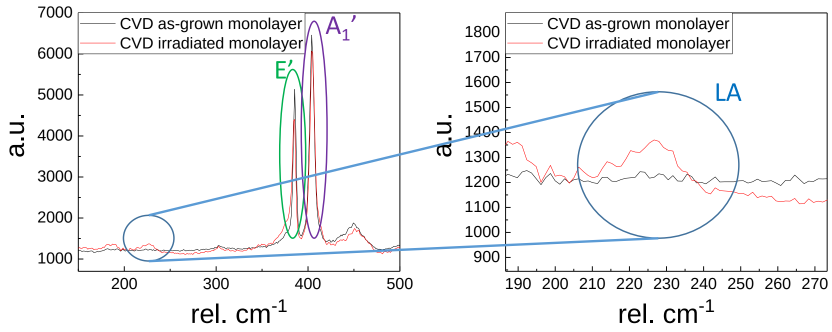

Figure SI2. A)Raman espectra for an as-grown and an irradiated MoS_2 monolayers. B) Zoom-in in the Raman spectra of panel a) where we can observe the emerging peak LA with higher defect densities.

It is worth noting that the LA peak is extremely difficult to identify in our as-grown samples due to their high crystallinity. However, it is possible to distinguish it from the background noise by averaging iterated spectra.

The intensity of this new peak is related to the intensity of each of the other two peaks as $\frac{I(LA)}{I(X)} = \frac{C(X)}{<l_D>^2}$, with X corresponding to peaks E' or A_1', and C a constant depending on defect size and laser characteristics.

On a former work[2] we derived the values of C(E') = 0.235 and C(A_1') =0.125 for MoS_2 membrane irradiated under the same conditions as those used in this work.

We irradiated samples of MoS_2 deposited on Transmission Electron Microscopy (TEM) grids in the same conditions used here and studied them by HR-TEM.

In this work we have used these relations to quantify the defect density of the samples. HR-STEM images also showed that most of the defects created during irradiation were single S vacancies (80%) with smaller densities of double S vacancies (17%) and Mo vacancies (3%). An example of these images can be found in Figure SI3.

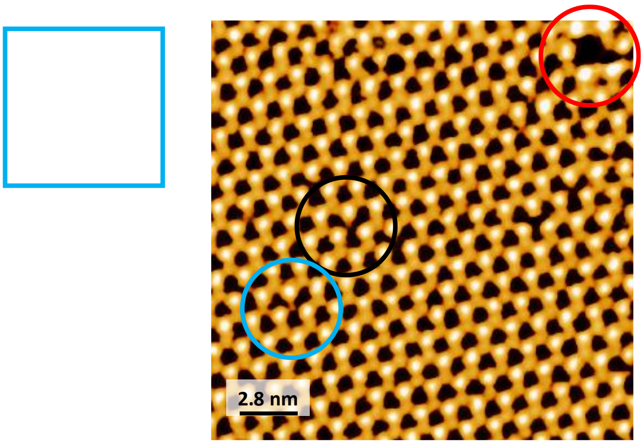

Figure SI3. Left: Detail of a HR-STEM image of an Ar^+ irradiated sample. Right: zoom showing a single S vacancy (blue) double S vacancy (black) and Mo vacancy (red).

Supplementary Information 3: Elastic characterization by nanoindentation prior to fatigue testing

We performed indentation experiments prior to fatigue testing with an AFM tip at the center of the suspended graphene membrane. Indentation curves were performed with a constant rate of 150nm/s up to a force close to the failure point. Our force (F) vs. indentation (δ) curves showed a cubic dependence, fitting the expression:[5,6]

$$F(\delta) = \pi T\delta + \frac{E}{a^2}\delta^3 \quad \text{equation 1}$$

where T is the pretension accumulated in the sheet, E is the elastic modulus of the membrane and a is its radius. From these measurements we derived average values of E=200 N/m and T=0.15 N/m.[2,7]

We also discarded poorly anchored membranes due to low adhesion to the substrate. For this purpose, we indented the membranes again up to a force close to the failure point and discarded those that showed hysteric behavior as the reported in figure SI4.

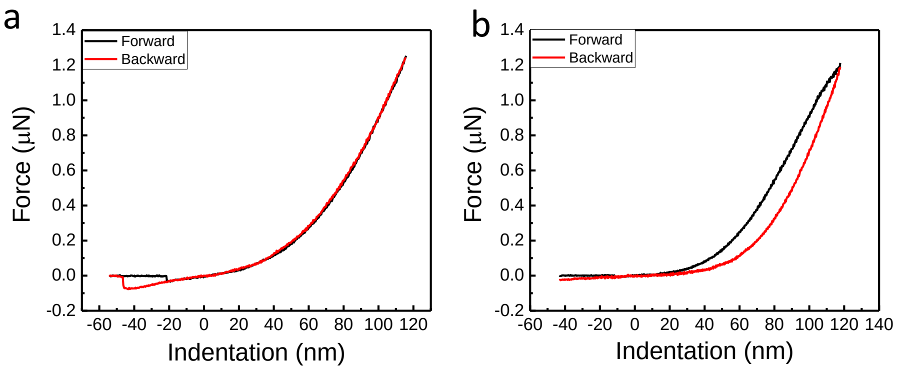

Figure SI4. a) Indentation curve on a well anchored microdrum. The deformation of the membrane shows no hysteretic behavior. b) indentation curve aquired on a membrane that slips through the substrate. Loading and unloading curves are different.

Supplementary Information 4: Fatigue protocol

All the fatigue measurements were carried out in a homemade AFM, controlled with WSxM software.[8] We used low-wear tungsten carbide-coated probes with tip radii ranging between 60 and 120 nm and nominal spring constant of 40 nN/nm, supplied by Team Nanotec.[9] These probes provide a uniform stress and avoid the effect of splinters that may appear on diamond tips. We found that low-wear tips with larger radii exhibited greater stability during fatigue testing. The spring constant of each cantilever was calibrated by using the Sader method[10,11] and each tip radius was recalibrated by imaging carbon nanotubes on a SiO_2 surface. We indented several micro drums of each defect density until fracture. With these indentations we evaluated the fracture strength for each defect density and applied the Weibull statistics to check the homogeneity of the samples. All the AC measurements presented here were obtained at 100 KHz. Previous tests at varying frequencies (between 50 kHz and 200 kHz) showed no dependence on the used frequency.

The process to apply an oscillating fatigue load is as follows:

- The force-deflection relation was calibrated by performing an indentation on the rigid SiO_2 substrate.
- We acquired non-invasive topographic images of the micro drums in non-contact mode and locating the AFM tip at the center of the selected micro drum.
- We performed an indentation at the center of the micro drum, not reaching fracture conditions. In the F-Z curve obtained with this indentation we calibrated the tip-sample displacement required to apply to induced the desired σ_{DC}. The indentation amplitude of the fatigues can also be calibrated from these curves.
- Then, we introduced the new value for z on the microscope and introduce the static load value to the feedback.
- We set the oscillation frequency at 100 kHz. This frequency is important as it is much lower than the resonance frequency of the cantilever and the micro drum, and far from resonances from the system. 100 kHz is also a frequency high enough to allow performing many load cycles in short periods of time.

All this process was monitored in real time with an oscilloscope showing the cantilever deflection (normal force).

Supplementary Information 5: Dependence of breaking strength with prestress of the samples

Suspended samples always present some level of residual stress, also known as prestress. It could affect to the evaluation of the breaking strength, and therefore to the evaluation of σ_{DC}/σ_F. To assess this issue, we performed F(δ) curves up to the fracture point where we evaluated the breaking strength and the tension of the membrane, T, according to expression:

$$F(\delta) = \pi T\delta + \frac{E}{a^2}\delta^3$$

Our data show no dependence of the breaking strength with the pre-tension of the membrane.

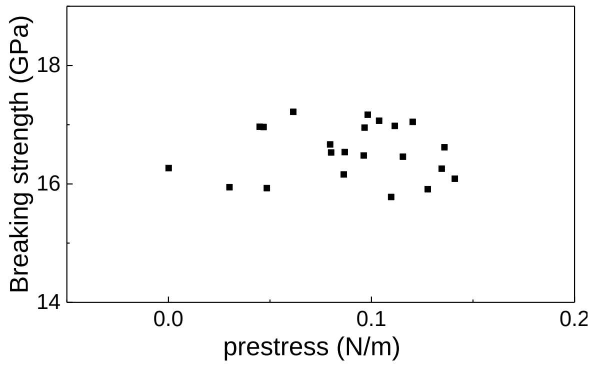

Figure SI5. The breaking strength is independent of the prestress in our samples.

Supplementary Information 6: Weibull distribution applied to nanoindentations

We studied the dispersion of the experimental values of the breaking strength of our samples using a Weibull distribution.[12] This probability density distribution has been widely used to characterize the failure of brittle materials with random defects. However, classical Weibul statistics for strength of solids do not properly apply at the nanoscale and further development was needed for its applicatin at the nanoscale. Pugno and Ruoff developed in 2006[13] a modification of Weibull statistics for describing the strength of solids at the nanoscale. This new approach, called nanoscale Weibul distribution, was developed to account for nearly defect-free structures and can be applied to treat samples with a small number of structural defects and assumes that just a very small defect can cause the failure of a nearly defect-free structure.

This Weibul distribution was already used in the seminal work on the intrinisic strengt of graphene[14] and has been aldo recenlty applied to evaluate the relaiability of graphene[15] and MoS_2.[16]

The nanoscale weibul accumulated failure probability at each strength (σ) is usually expresessed as:

$$P_F(\sigma) = 1 - e^{-n\left(\frac{\sigma}{\sigma_F}\right)^m},$$

where n is the number of critical defects at which the fracture starts.

Previous studies of nanoindentation on covalent 2D materails[2] have shown that fracture starts at the largest defect at the tip-sample contact area, where the maximum stress is applied. Therefore , the size of the largest defect under the AFM tip determines the breaking strength of the samples

For our samples the radius of the tip-sample contact area is well described by $R_c = R_{tip}\left(\frac{3F}{2\pi E_{2D} R_{tip}}\right)^{1/4}$,[5] where *F* the applied force E_{2D} is the two-diemnsional elastic modulus and R_{tip} is the radiou of the tip. For an AFM tip with a 30 nm radius applying a force of 500 nN, this expresion yields a contact radius of around 10 nm. Taking into account the defect density measured in our smaples, we calculate that we will find 1-3 critical defects at the tip-sample contact area. Although the strain is much higher right in the contact area than in the rest of the membrane, it is not uniform under the tip, but decreases with the distance to the apex.[17] This makes very unlikely to find two critical defects of the same size under the tip. Based on this estimation, we considered n=1 to analyse our data. Nonetheless, a different value of n (n=2,3) would only displace the center of our distribution from the average breaking strength.

A recent molecular dynamic study on realibity of MoS_2 have shown that *m*, the Weibull modulus in nanoscale MoS_2 is mainly dictated by defect density and configuration in the sample.[16] According to this simulations, defect engineering may be a viable route to increase consistency.[16] and the authors propose the creation of homogeneous defect type and density as a manner to decrease the variability of the strength from sample to sample and therefore to reduce the Weibull modulus. Our observation of increasing Weibull modulus with increasing defect density might seem, at a first glance, to contradict this theoretical prediction. However, this is not the case. The difference relies on the size of the sample that is strained compared to the mean distance between defects. If the size of the strained area is high enough to accommodate a high number of defects (>8), then, according to the authors, and only when combining samples with different defects configurations at each defect density, m will show a decreasing tendency with defect density. In general, m will increase with defect density owing to the strong effect of defect configuration. Due to the very localized nature of the applied load during nanoindentations, m measured by this technique is expected to decrease with defect density. Application of such large global strains to 2D membranes is still a challenge for the scientific community. After a thorough review of the published literature, we found only one publication describing an experimental setup that allows the application of global strains high enough to allow fracture.[18] The proposed setup is unique, involves many and difficult fabrication steps, and provides very low yield of successful devices.

Supplementary Information 7: Comparison of fatigue response of MoS_2 and graphene

Here we aim to compare the fatigue lifetime of single layer MoS_2 obtained in this work to the data obtained by Cui et al. for graphene samples in reference.[15] With this purpose we selected our results on MoS_2 ant those reported by Cui et al for graphene under similar conditions. **Figure SI3a** gathers data for σ_{AC}= 0.031-0.046 $\langle\sigma_F\rangle$.

Figure SI3b shows the comparison at varying σ_{AC} for micro drums supporting a σ_{DC}=0.6 <σ_F>. For an easier comparison, we converted our data to the units shown in ref.[15] We can see that the data for both covalent materials present similar values and trends.

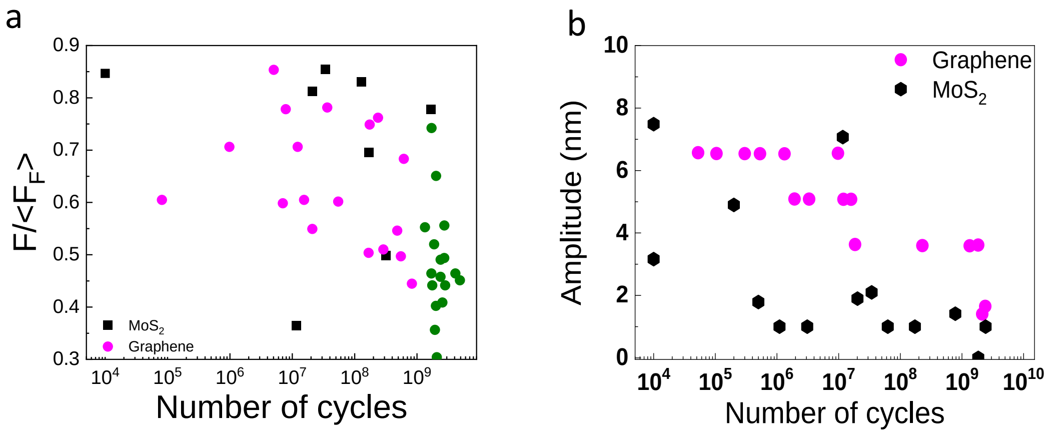

Figure SI6. a) F-N plots for as-grown MoS_2 micro drums (black) and graphene samples reported in ref[15] *(pink) . Green dots are those that did not break in neither batch of samples. b) A-N plots of our, as-grown MoS_2 micro drums (black) and the graphene samples reported in ref*[15] *(pink) for a constant static load of 0.36 <F_F>.*

Supplementary Information 8: Scanning Electron Microcopy images of MoS_2 membranes fractured by fatigue

We acquired Scanning Electron Microscopy (SEM) images of our membrane after failure by fatigue testing. These images showed all the MoS_2 drumheads broke catastrophically. We observed that tear started at the center of the suspended are, where the tip exerted the maximum force, and grow along crystallographic direction towards the walls of the circular wells reaching microns lengths.

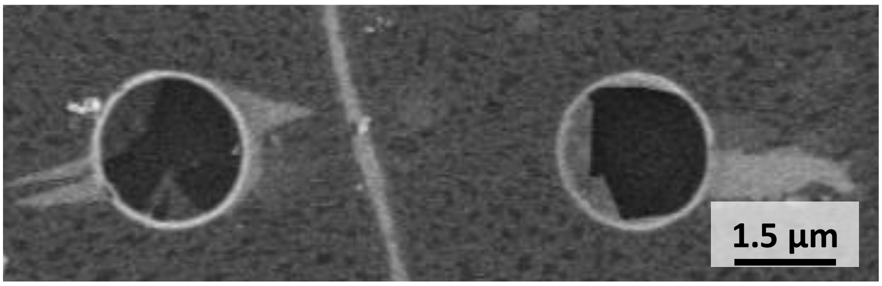

Figure SI7. SEM image of two representative membranes after failure by fatigue testing

Supplementary Information 9: Complementary S-N plots

The number of cycles that MoS_2 single layers with different defect densities can withstand at higher static loads and amplitudes follows a similar trend as for medium static and dynamic loads (shown in the main text) but with shorter times. **Figure SI8a** shows the number of cycles that the micro drums can withstand with a static load of 0.6 <σ_F>. In **Figure SI8b** we can see also see the number of cycles that the micro drums withstand with an amplitude as high as 0.125 <σ_F> and varying static load. We can see

that the behavior of the samples under fatigue is similar to that obtained at small amplitudes but with a much higher dispersion.

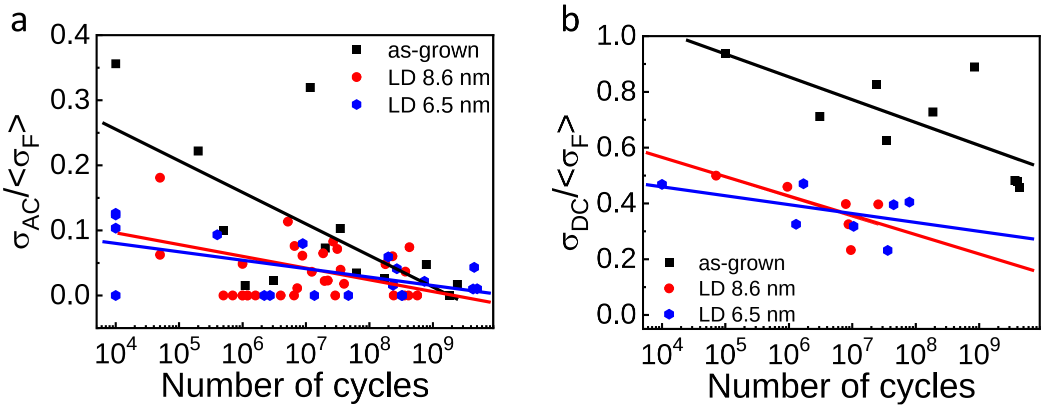

Figure SI8. a) Number of cycles that as-grown (black), irradiated with LD 8.6 nm (red) and irradiated with LD 6.5 nm (blue) single layer MoS_2 micro drums can withstand with a static load of 0.6 $<\sigma_F>$ and variable load amplitude. b) Number of cycles that as-grown (black), irradiated with LD 8.6 nm (red) and irradiated with LD 6.5 nm (blue) single layer MoS_2 micro drums can withstand with a loading amplitude of 0.125 $<\sigma_F>$ and variable static load.

Supplementary Information 10: Lateral Force Microscopy details

LFM images were acquired in a homebuilt AFM under vacuum condition with a base pressure of $5x10^6$mbar. We used AFM probes HQ-CSC38 Al/BS from MikroMasch with a force constant 0,03N/m. Images were acquired with a normal force of 1nN. We found that under these conditions we obtained improved resolution. For our experiments, prior to high-resolution imaging we scanned the membrane with an AFM tip at low loading forces during a period of 2 hours to remove adsorbates.

(1) Splendiani, A.; Sun, L.; Zhang, Y.; Li, T.; Kim, J.; Chim, C.-Y.; Galli, G.; Wang, F. Emerging Photoluminescence in Monolayer MoS2. *Nano Lett.* **2010**, *10* (4), 1271–1275. https://doi.org/10.1021/nl903868w.

(2) Manzanares-Negro, Y.; López-Polín, G.; Fujisawa, K.; Zhang, T.; Zhang, F.; Kahn, E.; Perea-López, N.; Terrones, M.; Gómez-Herrero, J.; Gómez-Navarro, C. Confined Crack Propagation in MoS2 Monolayers by Creating Atomic Vacancies. *ACS Nano* **2021**, *15* (1), 1210–1216. https://doi.org/10.1021/acsnano.0c08235.

(3) Fujisawa, K.; Carvalho, B. R.; Zhang, T.; Perea-López, N.; Lin, Z.; Carozo, V.; Ramos, S. L. L. M.; Kahn, E.; Bolotsky, A.; Liu, H.; Elías, A. L.; Terrones, M. Quantification and Healing of Defects in Atomically Thin Molybdenum Disulfide: Beyond the Controlled Creation of Atomic Defects. *ACS Nano* **2021**, *15* (6), 9658–9669. https://doi.org/10.1021/acsnano.0c10897.

(4) Mignuzzi, S.; Pollard, A. J.; Bonini, N.; Brennan, B.; Gilmore, I. S.; Pimenta, M. A.; Richards, D.; Roy, D. Effect of Disorder on Raman Scattering of Single-Layer Mo S 2. *Phys. Rev. B* **2015**, *91* (19), 195411. https://doi.org/10.1103/PhysRevB.91.195411.

(5) Begley, M. R.; Mackin, T. J. Spherical Indentation of Freestanding Circular Thin Films in the Membrane Regime. *Journal of the Mechanics and Physics of Solids* **2004**, *52* (9), 2005–2023. https://doi.org/10.1016/j.jmps.2004.03.002.
(6) Komaragiri, U.; Begley, M. R.; Simmonds, J. G. The Mechanical Response of Freestanding Circular Elastic Films Under Point and Pressure Loads. *Journal of Applied Mechanics* **2005**, *72* (2), 203–212. https://doi.org/10.1115/1.1827246.
(7) Chandler, T. G. J.; Vella, D. Indentation of Suspended Two-Dimensional Solids: The Signatures of Geometrical and Material Nonlinearity. *Journal of the Mechanics and Physics of Solids* **2020**, *144*, 104109. https://doi.org/10.1016/j.jmps.2020.104109.
(8) Horcas, I.; Fernández, R.; Gómez-Rodríguez, J. M.; Colchero, J.; Gómez-Herrero, J.; Baro, A. M. WSXM: A Software for Scanning Probe Microscopy and a Tool for Nanotechnology. *Review of Scientific Instruments* **2007**, *78* (1), 013705. https://doi.org/10.1063/1.2432410.
(9) *Hemisperical Cone Shaped Tip overview*. https://www.team-nanotec.de/index.cfm?contentid=10&shopAction=showProductDetails&id=325 (accessed 2023-03-07).
(10) Sader, J. E. Frequency Response of Cantilever Beams Immersed in Viscous Fluids with Applications to the Atomic Force Microscope. *Journal of Applied Physics* **1998**, *84* (1), 64–76. https://doi.org/10.1063/1.368002.
(11) Sader, J. E.; Larson, I.; Mulvaney, P.; White, L. R. Method for the Calibration of Atomic Force Microscope Cantilevers. *Review of Scientific Instruments* **1995**, *66* (7), 3789–3798. https://doi.org/10.1063/1.1145439.
(12) Lawn, B. Fracture of Brittle Solids. *(No Title)* **1993**, 194. https://doi.org/10.1017/cbo9780511623127.
(13) Pugno, N. M.; Ruoff, R. S. Nanoscale Weibull Statistics. *Journal of Applied Physics* **2006**, *99* (2), 024301. https://doi.org/10.1063/1.2158491.
(14) Lee, C.; Wei, X.; Kysar, J. W.; Hone, J. Measurement of the Elastic Properties and Intrinsic Strength of Monolayer Graphene. *Science* **2008**, *321* (5887), 385–388. https://doi.org/10.1126/science.1157996.
(15) Cui, T.; Mukherjee, S.; Sudeep, P. M.; Colas, G.; Najafi, F.; Tam, J.; Ajayan, P. M.; Singh, C. V.; Sun, Y.; Filleter, T. Fatigue of Graphene. *Nature Materials* **2020**, *19* (4), 405–411. https://doi.org/10.1038/s41563-019-0586-y.
(16) Cui, T.; Mukherjee, S.; Onodera, M.; Wang, G.; Kumral, B.; Islam, A.; Shayegannia, M.; Krishnan, G.; Barri, N.; Serles, P.; Zhang, X.; Sassi, L. M.; Tam, J.; Bassim, N.; Kherani, N. P.; Ajayan, P. M.; Machida, T.; Singh, C. V.; Sun, Y.; Filleter, T. Mechanical Reliability of Monolayer MoS2 and WSe2. *Matter* **2022**, *5* (9), 2975–2989. https://doi.org/10.1016/j.matt.2022.06.014.
(17) López-Polín, G.; Jaafar, M.; Guinea, F.; Roldán, R.; Gómez-Navarro, C.; Gómez-Herrero, J. The Influence of Strain on the Elastic Constants of Graphene. *Carbon* **2017**, *124*, 42–48. https://doi.org/10.1016/j.carbon.2017.08.023.
(18) Pérez Garza, H. H.; Kievit, E. W.; Schneider, G. F.; Staufer, U. Controlled, Reversible, and Nondestructive Generation of Uniaxial Extreme Strains (>10%) in Graphene. *Nano Lett* **2014**, *14* (7), 4107–4113. https://doi.org/10.1021/nl5016848.
